# CESNET-TLS-Year22: A year-spanning TLS network traffic dataset from backbone lines

**DOI:** 10.1038/s41597-024-03927-4

**Published:** 2024-10-18

**Authors:** Karel Hynek, Jan Luxemburk, Jaroslav Pešek, Tomáš Čejka, Pavel Šiška

**Affiliations:** 1 CESNET, Prague, Czech Republic; 2 Faculty of Information Technology, Czech Technical University in Prague, Prague, Czech Republic

**Keywords:** Information technology, Scientific data

## Abstract

The modern approach for network traffic classification (TC), which is an important part of operating and securing networks, is to use machine learning (ML) models that are able to learn intricate relationships between traffic characteristics and communicating applications. A crucial prerequisite is having representative datasets. However, datasets collected from real production networks are not being published in sufficient numbers. Thus, this paper presents a novel dataset, CESNET-TLS-Year22, that captures the evolution of TLS traffic in an ISP network over a year. The dataset contains 180 web service labels and standard TC features, such as packet sequences. The unique year-long time span enables comprehensive evaluation of TC models and assessment of their robustness in the face of the ever-changing environment of production networks.

## Background & Summary

The fraction of encrypted Internet traffic is increasing, and thus, the traditional payload-based systems for traffic classification and threat detection are becoming obsolete. A promising solution is machine learning (ML) models utilizing sequences of packet sizes, times, and other connection metadata statistics that are available even when the traffic is encrypted. However, compared to different ML sub-fields, such as computer vision or natural language processing, advancements in ML models for network traffic classification are being slowed down because of a lack of large representative datasets collected from real operational networks. Commercial organizations are reluctant to share such data because of its business value^1^, whereas universities and other research institutions often lack access to networks with diverse traffic. Moreover, existing public datasets tend to have a limited time span or do not include date-time information about samples at all. Having samples distributed in time is crucial for measuring how robust ML models are in the face of evolving network traffic–emerging new applications, protocol updates, and class imbalance changes.

As a follow-up of previous CESNET traffic classification datasets CESNET-TLS22^2^ and CESNET-QUIC22^3^, this paper introduces a new long-spanning TLS dataset called CESNET-TLS-Year22^4^. It was captured over the entire year of 2022 at the backbone 100 Gbps lines of the CESNET3 network, which is an ISP network with around half a million users. The data are provided in the form of network flows representing TLS communications extended with packet sequences describing the first 30 packets of the connection, packet histograms, and fields extracted from the TLS ClientHello message. Altogether, the dataset contains 180 different labels (selected web services) that are split into 24 traffic categories.

There are no public traffic classification datasets of comparable size or time span. The most similar datasets are CESNET-QUIC22^3^ and AppClassNet^1^. CESNET-QUIC22 focuses on the QUIC protocol, has 102 classes, and spans one month. AppClassNet consists of both TCP and UDP traffic, was labeled with a commercial-grade DPI system with 500 classes in total, but its data went through an extensive transformation process to remove business-sensitive information (such transformation was not required in our case). Also, the AppClassNet dataset does not include date-time features, which makes it unusable for time-aware evaluation. Evaluation of network traffic classifiers should be time-consistent in the sense that test data have to come after train data. Not respecting the time order can lead to results ten-percent higher than the actual time-consistent results, as was demonstrated, for example, in Android malware classification^5^. Our new CESNET-TLS-Year22^4^ dataset provides data over an entire year, which enables time-aware evaluation on multiple testing periods subsequent to the model training period (an example is showcased in the Data Drift Analysis section). The dataset is thus suitable for measuring how stable a classifier is in time, for researching different model retraining strategies, and for comprehensive evaluation of traffic classifiers overall.

Moreover, recent related works proposed various solutions to the ever-changing network environment. For example, there are advancements in incremental class learning^6^ that focuses on how to add new classes to existing models without retraining them from scratch, or few-shot learning^7,8^ that aims to extract knowledge from a set of training tasks in order to better perform on underrepresented classes (e.g., when a limited number of samples is available for new applications). These novel approaches will benefit from the long time span of CESNET-TLS-Year22^4^ and the fact that it was captured in a real operational network.

## Methods

**Fig. 1:**
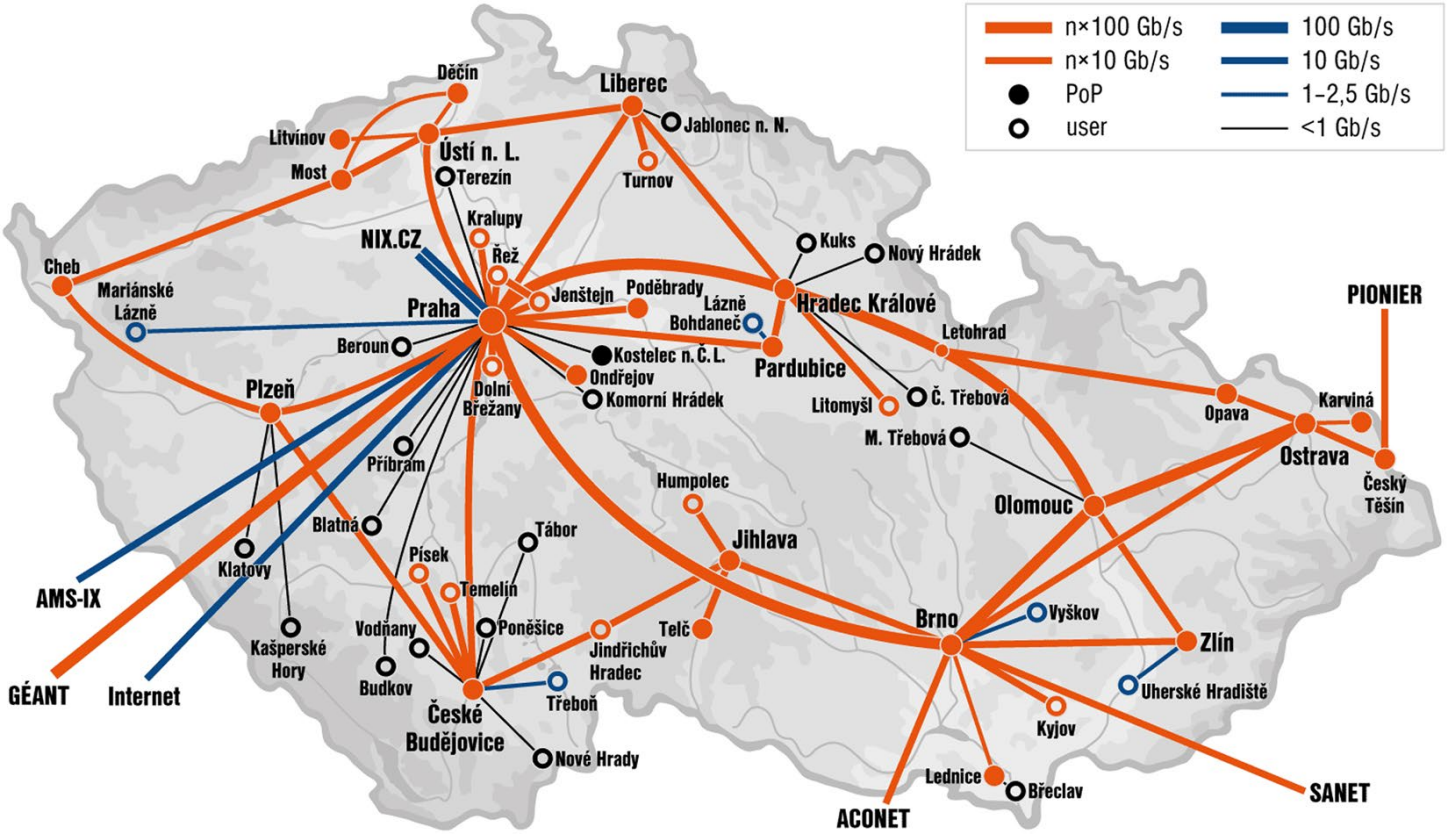
The topology of the CESNET3 network.

The creation of the CESNET-TLS-Year22^4^ dataset follows a similar process to that used in our earlier work on the CESNET-QUIC22 dataset. We previously published much of the methodology in the data article^3^, but for convenience, we will reiterate the same information in this work. The CESNET-TLS-Year22^4^ dataset has been captured at monitoring vantage points located at the perimeter of the CESNET3 network–a national research and education network in the Czech Republic that provides internet access to around half a million users. CESNET3 network spans the whole Czech Republic and connects large public institutions such as universities, research institutions, campuses, hospitals, and municipal offices. Its topology is shown in Fig. 1.

### Ethics statement

The privacy of the CESNET3 network users is a fundamental concern in our work, leading us to conduct our research with careful consideration. The indisputable advantages of real traffic generated by hundreds of thousands of people come with understandable privacy concerns. Thus, we used only automatic data processing with immediate data anonymization. With this, we declare that we did not analyze or manually process non-anonymized data or perform any procedures that could allow us to track users or reveal their identities.

The publication of the dataset has been approved by the Committee for Ethics in Research of the Scientific Council of the Czech Technical University in Prague under reference number 0000-07/24/51902/EKČVUT. The approval also includes a waiver of explicit user consent for publishing the dataset since the data are completely anonymous, and it is impossible to trace the identity of the data subjects. Moreover, all users of the CESNET3 network agreed with the terms and conditions that define a monitoring process for optimization and improvement of services (including related research) and allow sharing of the data with third parties after anonymization (https://www.cesnet.cz/en/gdpr).

### Data collection process

The data collection process utilized the CESNET3 monitoring infrastructure, which follows the traditional flow-based design as described by Hofstede *et al*.^9^. Five network probes are distributed across multiple geographic locations (Prague, Brno, and Ostrava), and each monitors one or multiple 100 Gbps peering lines via passive optical TAPs or border router SPAN ports. Flow data are then transmitted to a single flow collector, where the data are processed and stored. System clocks of the network probes are synchronized using the NTP protocol to ensure time-consistent features.

The dataset creation workflow can be divided into five stages: (1) Service Selection, (2) Flow Enrichment and Export, (3) Flow Collection and Filtration, (4) Flow Sampling, and (5) Data Anonymization and Curation. The workflow is visualized in Fig. 3, and the stages are described in the following sections.

**Fig. 2:**
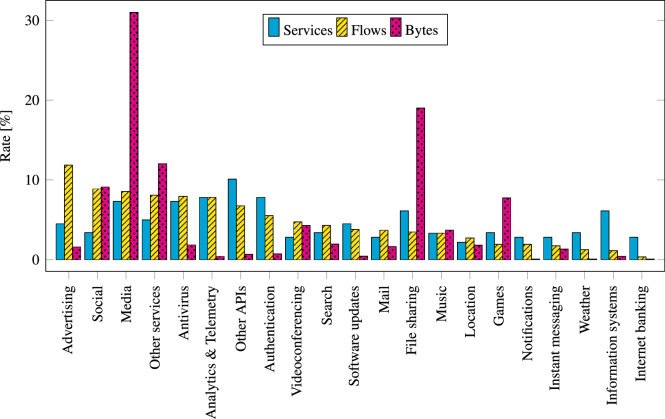
The breakdown of dataset traffic into categories, showing fractions of services, bytes, and flows.

**Fig. 3:**
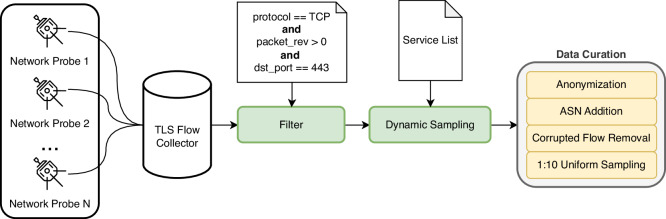
The illustrative scheme of dataset creation workflow.

### Service selection

To create an authentic dataset representing the real network environment and to collect an ample amount of flow data for each service required us to make a deliberate selection of the services to be included in the dataset. We selected the services based on the following criteria:

#### Traffic volume

We prioritized services with larger traffic volumes so that the dataset covers a substantial amount of TLS traffic of the CESNET3 network (45% of all TLS traffic in the network is covered with the selected services).

#### Diversity

The diversity of the dataset’s services is essential to capture various types of traffic and to build a representative dataset.

The dataset contains 180 different web services, each representing its own class that can be used in network traffic classification tasks. The selected web services can be divided into 24 categories that are listed in Fig. 2, which also shows fractions of services, bytes, and flows per category. The services were recognized using the SNI domain transmitted in the ClientHello message of the TLS handshake. To find the domains associated with a particular service, we searched its online documentation, used Netify’s Application Lookup Tool (https://www.netify.ai/resources/applications), or handpicked domains from the observable domains in the CESNET3 network.

### Flow enrichment and export

Each network probe was installed with ipfixprobe (https://github.com/CESNET/ipfixprobe), which is a high-performance bidirectional flow exporter capable of processing 100 Gbps traffic while exporting extended flow features. During the dataset collection period, the entire year of 2022, ipfixprobe has been kept updated, which also influenced the resulting data (more information is provided in the Technical Validation section). We used ipfixprobe’s TLS plugin, which effectively parses TLS handshakes. When the TLS plugin detects a TLS connection handshake, it enriches the flow with the SNI domain transmitted inside the ClientHello message. Moreover, we also used the PSTATS plugin, which exports metadata statistics (size, direction, and inter-packet time) about the first 30 transmitted data packets, and the PHIST plugin, which exports histograms of packet sizes and inter-packet times of the entire flow.

The flow exporter was set with 5 minutes of active timeout and 65 seconds of inactive timeout. Flows describing long connections are exported when the active timeout of 5 minutes is reached, even though the actual connection has not yet ended. A connection is considered idle, and its flow is exported when no packet belonging to that connection is observed within the inactive period of 65 seconds. Exported flows from each network probe are then transmitted with the IPFIX protocol^10^ to a single collector, where additional processing is performed.

### Flow collection and filtration

Flows from all network probes are collected using the ipfixcol2 (https://github.com/CESNET/ipfixcol2) flow collector, which was executed with configuration to receive TLS flows enriched with the SNI domain. All received data were converted using ipfixcol2 into the NEMEA framework^11^, which provides efficient stream-based flow processing. We performed flow filtration using the NEMEA filtering module (https://github.com/CESNET/Nemea-Modules/). This module selected TLS flows that had destination port 443/TCP and had at least one packet in both directions to filter unidirectional flows. Unidirectional flows can be formed in the network due to service scanning, connection errors, or other network phenomena such as asymmetric routing. Bidirectional TLS flows with the SNI domain were passed to the following sampling stage.

### Flow sampling

Since our goal is long-term flow capture from a large backbone network, we must use sampling to maintain a reasonable dataset size. We decided to use a dynamic sampling ratio for each service to soften the class imbalances in the dataset. Each service is sampled at a different rate, depending on the amount of traffic it generates. For this purpose, we used the TLS SNI dataset saver NEMEA module, which is capable of online flow sampling based on the prevalence of the corresponding service. A handful of TLS services, mainly operated by large technological companies (Google, Meta, Apple, and Microsoft), generate the majority of TLS traffic on the CESNET3 network, and the dynamic sampling strategy ensures that even minority classes are represented with a sufficient amount of flows. We sorted the services based on the amount of traffic. The top 5% of the most prevalent services were sampled in the 1:15 ratio, and the bottom 60% of services were not sampled at all. The remaining 35% were sampled in a ratio ranging between 1:2 and 1:9, depending on their prevalence. The amount of traffic for each service was continuously monitored during the capture, and its sampling ratio was updated every five minutes.

### Data anonymization and curation

To protect the privacy of CESNET3 users, we transformed client IP addresses using the SHA hash function with a random secret (salt). We omitted other fields that could lead to user identification, such as source transport ports or MAC addresses. Moreover, we rounded the exact timestamps–the start times of all flows are clipped to the start of an hour, and the end times are adjusted to maintain the original flow durations. We opted to proceed with this anonymization procedure instead of simple IP address removal to enable traffic classification approaches that require flow aggregation by source. The hashing of client addresses, removing source ports, and concealing the exact timestamps break the link between a flow and the actual user; thus, user identification is impossible.

After completing the anonymization procedure, we enriched flow data with the destination autonomous system number (ASN). We map destination (i.e., server) IP addresses to ASNs. Moreover, we cleaned the dataset of corrupted flows. We also removed flows that contained less than three data packets since this is a minimal number of packets for a successful TLS handshake. These short flows emerge on ISP networks from unstable links in user networks (e.g., long distances from WiFi routers) or from application scans. Therefore, we consider these flows as noise. The last curation step was uniform 1:10 sampling of all the data, which was necessary to maintain a reasonable dataset size.

## Data Records

The data records and the structure of the CESNET-TLS-Year22^4^ dataset are similar to our previous works^2,3^. CESNET-TLS-Year22 consists of network flows describing encrypted TLS communication and is available for download on the Zenodo platform^4^. Flows in the dataset are extended with packet sequences, histograms, and fields extracted from the TLS ClientHello message, which is transmitted in the first packet of the TLS connection handshake. The most important extracted handshake field is the SNI domain, which is used for ground-truth labeling. The following sections describe two types of data features–packet sequences, which provide information about the first 30 packets of a connection, and flow statistics describing the entire connection.

### Packet sequences

Sequences of packet sizes, directions, and inter-packet times are standard data input for traffic analysis. For packet sizes, we consider the payload size after transport headers (TCP headers for the TLS case). We omit packets with no TCP payload, for example ACKs, because zero-payload packets are related to the transport layer internals rather than services’ behavior. Packet directions are encoded as ±1, where “+1” means a packet sent from client to server and “−1” a packet from server to client. Inter-packet times depend on the location of communicating hosts, their distance, and on the network conditions on the path. However, it is still possible to extract relevant information that correlates with user interactions and, for example, with the time required for an API/server/database to process the received data and generate a response. Packet sequences have a maximum length of 30, which is the default setting of the used flow exporter. We also derive three fields from each packet sequence: its length, time duration, and the number of roundtrips. The roundtrips are counted as the number of changes in the communication direction; i.e., each client request and server response pair counts as one roundtrip.

### Flow statistics

Each data record also includes standard flow statistics, representing aggregated information about the entire bidirectional connection. The fields are the number of transmitted bytes and packets in both directions, the duration of the flow, and packet histograms. The packet histograms include binned counts (not limited to the first 30 packets) of packet sizes and inter-packet times in both directions. There are eight bins with a logarithmic scale; the intervals are 0–15, 16–31, 32–63, 64–127, 128–255, 256–511, 512–1024, >1024 [ms or B]. The units are milliseconds for inter-packet times and bytes for packet sizes. For more information about the PHISTS plugin, please refer to the ipfixprobe documentation. Moreover, each flow has its end reason–either it ended with the TCP connection termination (FIN packets), was idle, reached the active timeout, or ended due to other reasons. This corresponds with the official IANA IPFIX-specified values^12^. The FLOW_ENDREASON_OTHER field represents the forced end and lack of resources reasons. The distribution of selected data features is visualized in Fig. 7.

**Fig. 4:**
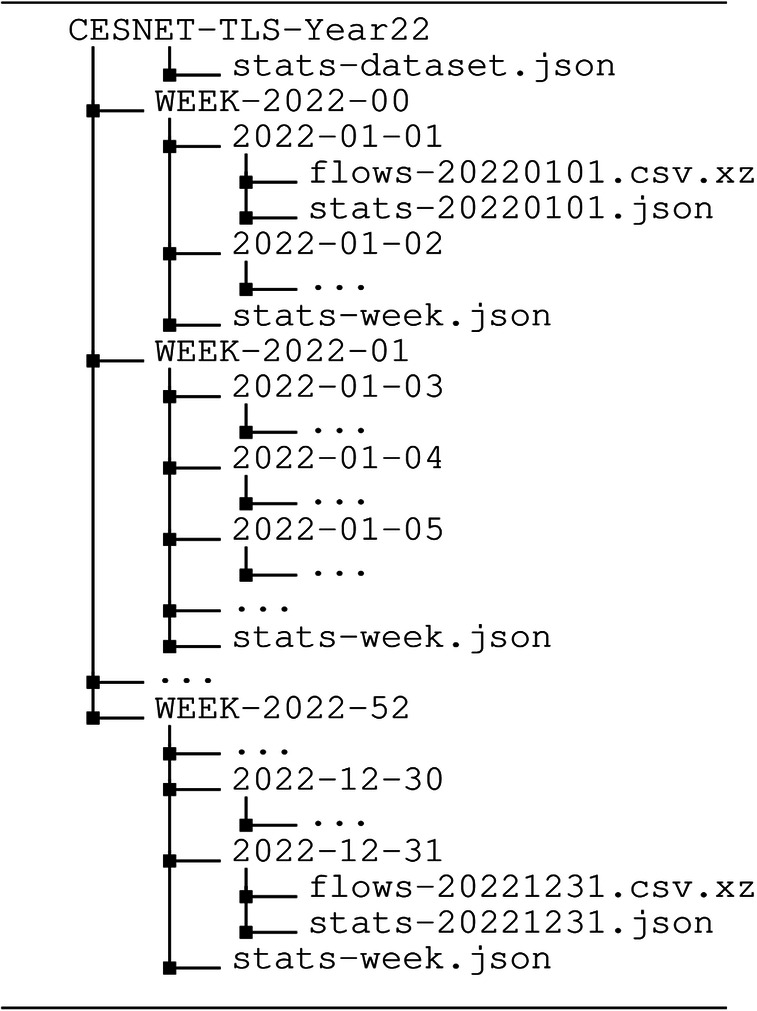
The file structure of the CESNET-TLS-Year22 dataset.

**Fig. 5:**
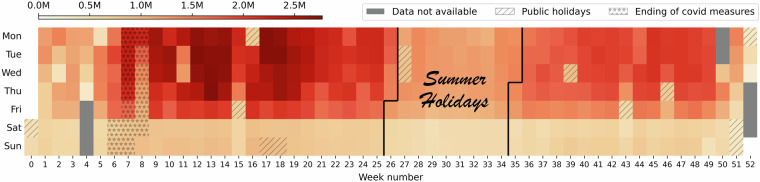
The heatmap of the number of flows throughout the year 2022.

**Fig. 6:**
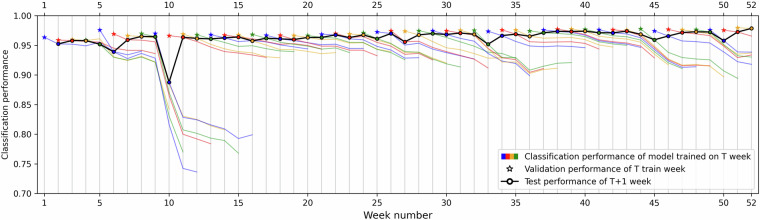
The classification performance of a neural network trained in each of 52 weeks in the dataset.

### Dataset structure

The dataset flows are delivered in compressed CSV files that are organized per week and date, as shown in Fig. 4. CSV files contain one flow per row; data columns are summarized in Table 2. For each flow data file, there is a JSON file with the total number of saved flows and the number of flows per service. There are also files aggregating flow counts for each week (stats-week.json) and for the entire dataset (stats-dataset.json).

**Table 1:** Software versions used for creating the dataset.

Name	Version	Link
ipfixprobe	4.0.0–4.7.1	https://github.com/CESNET/ipfixprobe
ipfixcol2	2.2.1	https://github.com/CESNET/ipfixcol2
NEMEA Framework	0.14.0	https://github.com/CESNET/Nemea-Framework
NEMEA modules	2.20.0	https://github.com/CESNET/Nemea-Modules

## Technical Validation

Technical validation of the dataset is threefold: (1) Validation of the Data Correctness, (2) Volumetric Analysis, and (3) Data Drift Analysis. The results of the technical validation are described in the following sections.

### Validation of data correctness

The data correctness validation aimed to recognize semantic mistakes in the data and to find samples with properties that should be impossible for valid TLS communications. We checked all flows for the following:

- Each flow is bidirectional and has at least three data packets, which is the minimum number of packets to complete the TLS handshake.
- The first data packet is transmitted from the client to the server, it includes the ClientHello message, and the SNI domain is parsed.
- The end time of each flow is never before its start time, and the inter-packet times are never negative. These time inconsistencies can arise in high-speed traffic processing due to, for example, hardware clock synchronization.
- For each flow, the number of entries in the histogram of packet sizes is one bigger than the number of entries in the histogram of inter-packet times.

The validation of data correctness was successful, and the dataset does not contain the aforementioned semantic errors.

### Volumetric analysis

The volumetric analysis aims to validate the relationship between the captured data volume and the real-world phenomena in the Czech Republic. The dataset per-day flow counts are shown as a heatmap in Fig. 5. The CESNET3 network connects public organizations such as universities, hospitals, and municipal offices. Thus, the connected users are students, researchers, and employees of the public sector, who mostly use the network during workdays. The heatmap clearly shows this pattern. Weekends and public holidays contain far less flow data than workdays. Moreover, public holidays that are close to a weekend are often surrounded by days with lower traffic volume due to employees’ vacations. These observations are in alignment with a previous study of the network^13^.

Since many of the connected organizations are universities, the heatmap also shows the academic year with traffic peaks in the semesters and periods of lower traffic volume during school holidays. We can see higher traffic volume during the summer semester (around weeks 7 to 20) compared to the winter semester (around weeks 38 to 50). We attribute this difference to the lifting of COVID-19 measures on February 19, 2022 (Saturday, week 7). Such change naturally resulted in increased activity since postponed in-person events that could not be organized during the pandemic took place at that time.

Apart from eight days during which the monitoring infrastructure experienced outages, we did not observe artifacts in the data. The volumetric analysis showed a strong relationship between captured data volume and the real-world events and phenomena occurring in the Czech Republic in 2022.

### Data drift analysis

The goal of this section is to validate the data and label distributions and to showcase an essential characteristic of network traffic–that it is undergoing constant change due to, for example, network congestions, new applications, and protocol updates. Therefore, when a classification model is trained on data collected in a particular training period, the model accuracy should be the best for test samples coming right after the training period, and a gradual drop in performance is expected as the time between the training period and test samples increases. This is because a powerful model, such as a deep neural network, learns intricate relationships between the network data and target labels; however, these relationships are often valid for a limited amount of time, after which the model becomes outdated and starts to make more mistakes.

An example of this phenomenon can be found in our previous dataset CESNET-QUIC22^3^ that contains data drift in the form of a modified size of the TLS certificate of Google services, which resulted in a steep drop in the classification performance^14^. In the following analysis, our goal is to look for similar data drift events in the CESNET-TLS-Year22^4^ dataset. As the authors of the dataset and maintainers of the used monitoring infrastructure, we are in the best position to provide explanations for discovered data drift events, which in turn should help future dataset users better understand the data. Moreover, as part of the technical validation process, we search the dataset for artificial drift events arising from software and hardware updates of the monitoring infrastructure rather than from natural changes in traffic characteristics.

#### Evolution of model classification performance

We base the data drift analysis on the classification performance of our established neural network architecture designed for processing network traffic that was published in our previous work^14^. A separate model was trained on the traffic of each week (week *T*) and then tested on the following seven weeks (weeks *T* + 1 to *T* + 8). The traffic of each week *T* was split between a training set and a validation set, which was used to measure the validation performance. This procedure was repeated for all 52 weeks in the dataset (except that for the last eight weeks, there are fewer testing weeks). The resulting model per-week accuracies are shown in Fig. 6. Results for *T* + 1 weeks are highlighted in the figure as we consider them the most relevant due to the shortest time gap between the training period and testing data. In accordance with our expectations, the figure shows that for most models, there is a slow, gradual decrease in their performance over the seven test weeks. The average of *T* + 1 week accuracies is 96.3%, which drops to 90.5% for *T* + 8 weeks. The average validation performance is 97.2%, which represents model performance without data drift because validation samples are from the same time period as the training samples.

Even though most performance changes were gradual, several events through the year 2022 had a significant effect on the dataset traffic characteristics and, therefore, on the measured performances. The first bigger drop occurred in week 6. On Monday of this week, 7.2.2022, a peering link with one of CESNET’s partners was reestablished after an almost two-month pause. The data from the new peering increased the share of Google services in the dataset, resulting in a step-change of the data and label distributions. Such changes in the network infrastructure are natural and should be present in the dataset. Other smaller performance drops can be seen in weeks 27 and 33, which correspond to the start and the end of the summer holidays (as seen in Fig. 5), for which the traffic properties of the CESNET3 network are expected to deviate from the usual work weeks due to employees’ vacations and closed universities. The same interpretation applies to the drop around week 44, as this is the time of fall holidays in the Czech Republic, and to the drop around week 49, which is right before the Christmas holidays. We consider all the aforementioned drops natural as they stem from regular traffic variations in the CESNET3 network.

However, the most significant performance drop appeared in week 10, in which the monitoring infrastructure was updated to increase its resilience against volumetric network attacks. After the Russian invasion of Ukraine on 24.2.2022, the CESNET3 network experienced a surge of DDoS attacks^15^. These attacks generated a large number of flows and overwhelmed the output bandwidth of network probes, which resulted in data loss. On Wednesday, 9.3.2022, of week 10, a new version of the ipfixprobe flow exporter was deployed to mitigate the data loss. One of the new features was skipping of retransmitted packets in packet sequences. This feature helped to reduce the bandwidth between network probes and the flow collector; however, it also changed the distribution of packet sequences, resulting in a steep drop in the model performance. This data drift cannot be considered natural. We thus recommend using the dataset in two separate periods, weeks 1–9 and weeks 11–52, in order to avoid training of models on traffic captured before the packet retransmission change while testing on traffic captured after the change was implemented. Nevertheless, week 10 can be used for designing robust models since such changes in the network monitoring infrastructure can happen.

## Usage Notes

The dataset is available in the form of CSV files on the Zenodo platform^4^. Apart from the CSV format, we also provide the dataset through our data handling Python toolset called cesnet-datazoo^16^. The toolset streamlines access to the CESNET-TLS-Year22^4^ dataset, provides flow data in multiple interfaces (Pandas DataFrames and Pytorch DataLoaders), and overall facilitates reproducible research. The cesnet-datazoo toolset (https://github.com/CESNET/cesnet-datazoo) is documented and can be installed from PyPI or GitHub. We have also prepared a collection of example Jupyter notebooks (https://github.com/CESNET/cesnet-tcexamples) that showcase the use of our traffic classification datasets, see month_evaluation_cesnet_tls_year22.ipynb for a notebook working with CESNET-TLS-Year22.

## Figures and Tables

**Fig. 7:**
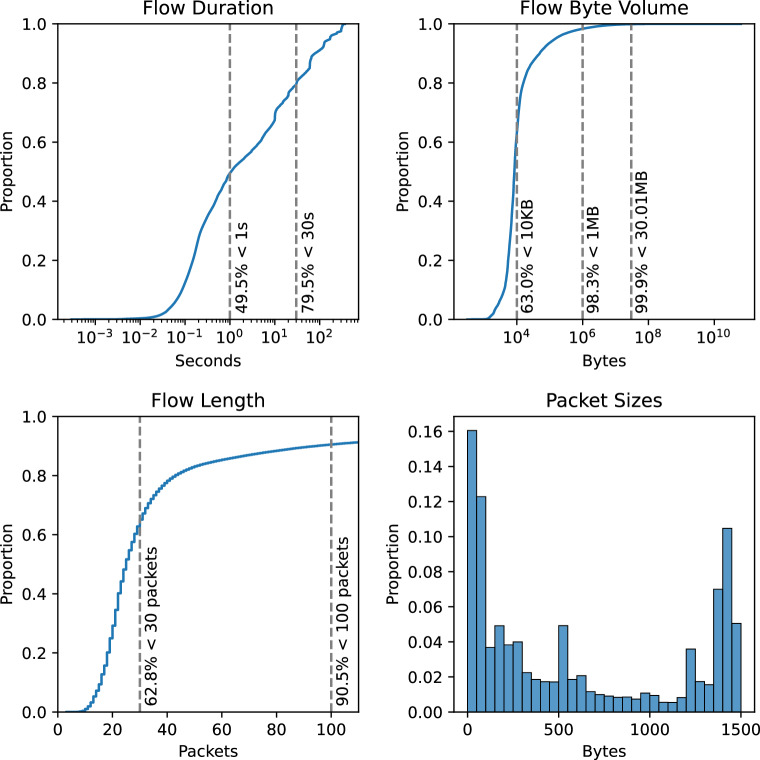
The distribution of selected data features.

## Data Availability

The dataset has been produced using open-source software. The flow exporter ipfixprobe, flow collector ipfixcol2, the NEMEA processing system, and its modules (unirec filter, sni dataset saver, and logger for CSV conversion) are available on GitHub. The versions of used software with links to corresponding repositories are summarized in Table 1.

## A Additional Figures

**Table 2:** The description of flow data fields in CSV files.

Column Name	Column Description
ID	Unique identifier
SRC_IP	Source IP address
DST_IP	Destination IP address
DST_ASN	Destination autonomous system number
SRC_PORT	Source port
DST_PORT	Destination port
PROTOCOL	Transport protocol^a^
FLAG_CWR	TCP CWR flag presence in client to server transmission
FLAG_CWR_REV	TCP CWR flag presence in server to client transmission
FLAG_ECE	TCP ECE flag presence in client to server transmission
FLAG_ECE_REV	TCP ECE flag presence in server to client transmission
FLAG_URG	TCP URG flag presence in client to server transmission
FLAG_URG_REV	TCP URG flag presence in server to client transmission
FLAG_ACK	TCP ACK flag presence in client to server transmission
FLAG_ACK_REV	TCP ACK flag presence in server to client transmission
FLAG_PSH	TCP PSH flag presence in client to server transmission
FLAG_PSH_REV	TCP PSH flag presence in server to client transmission
FLAG_RST	TCP RST flag presence in client to server transmission
FLAG_RST_REV	TCP RST flag presence in server to client transmission
FLAG_SYN	TCP SYN flag presence in client to server transmission
FLAG_SYN_REV	TCP SYN flag presence in server to client transmission
FLAG_FIN	TCP FIN flag presence in client to server transmission
FLAG_FIN_REV	TCP FIN flag presence in server to client transmission
TLS_SNI	Server Name Indication domain
TLS_JA3	JA3 fingerprint of TLS client
TIME_FIRST	Timestamp of the first packet in format YYYY-MM-DDTHH-MM-SS.ffffff
TIME_LAST	Timestamp of the last packet in format YYYY-MM-DDTHH-MM-SS.ffffff
DURATION	Duration of the flow in seconds
BYTES	Number of transmitted bytes from client to server
BYTES_REV	Number of transmitted bytes from server to client
PACKETS	Number of packets transmitted from client to server
PACKETS_REV	Number of packets transmitted from server to client
PPI ^b^	Packet sequence in the format: [[inter-packet times], [packet diretions], [packet sizes]]
PPI_LEN	Number of packets in the PPI sequence
PPI_DURATION	Duration of the PPI sequence in seconds
PPI_ROUNDTRIPS	Number of roundtrips in the PPI sequence
PHIST_SRC_SIZES	Histogram of packet sizes from client to server
PHIST_DST_SIZES	Histogram of packet sizes from server to client
PHIST_SRC_IPT	Histogram of inter-packet times from client to server
PHIST_DST_IPT	Histogram of inter-packet times from server to client
APP	Web service label
CATEGORY	Service category
FLOW_ENDREASON_IDLE	Flow was terminated because it was idle
FLOW_ENDREASON_ACTIVE	Flow was terminated because it reached the active timeout
FLOW_ENDREASON_END	Flow ended with the TCP connection termination
FLOW_ENDREASON_OTHER	Flow was terminated for other reasons

^a^TLS uses TCP as the transport protocol.

^b^PPI in field names stands for per-packet information, which is another common name for the packet sequences data.
