# Author Correction: CESNET-TLS-Year22: A year-spanning TLS network traffic dataset from backbone lines

**DOI:** 10.1038/s41597-024-04055-9

**Published:** 2024-11-05

**Authors:** Karel Hynek, Jan Luxemburk, Jaroslav Pešek, Tomáš Čejka, Pavel Šiška

**Affiliations:** 1 CESNET, Prague, Czech Republic; 2 Faculty of Information Technology, Czech Technical University in Prague, Prague, Czech Republic

**Keywords:** Information technology, Scientific data, Information technology, Scientific data

Correction to: *Scientific Data* 10.1038/s41597-024-03927-4, published online 18 October 2024

In this article in the contributing author section the initials P.Å should have been P. Š. The original article has been corrected.

